# Functional Characterization of Pomegranate CAMTA3 in Cold Stress Responses

**DOI:** 10.3390/plants14050813

**Published:** 2025-03-05

**Authors:** Shuangshuang Zhao, Rui Lu, Lijuan Feng, Mengyu Zheng, Han Zhang, Yanlei Yin, Ling Zheng

**Affiliations:** 1 Shandong Provincial Key Laboratory of Plant Stress, Life Science College, Shandong Normal University, Jinan 250014, China; zhaoshuangqw12@163.com (S.Z.); 15552497741@163.com (R.L.); 13256195849@163.com (M.Z.); 17862508580@163.com (H.Z.); 2 Shandong Institute of Pomology, Taian 271000, China; fenglj1230@126.com (L.F.); yylei66@sina.com (Y.Y.)

**Keywords:** pomegranate, cold stress, *CAMTA* genes

## Abstract

Cold stress is a significant factor limiting plant growth and development. Pomegranate is particularly susceptible to low temperatures. Calmodulin-binding transcriptional activators (CAMTAs) are key regulators of cold stress tolerance in plants. In this study, we conducted a comprehensive analysis of the CAMTA family proteins across 12 species, including *Punica granatum* (pomegranate), using bioinformatic methods. Pomegranate *CAMTA3* (*PgCAMTA3*) was isolated and characterized, and it demonstrated enhanced cold tolerance when expressed in *Arabidopsis thaliana*. Quantitative real-time PCR (qRT-PCR) analysis showed that the expression of *PgCAMTA3* was up-regulated under cold and ABA treatments in pomegranates. Two *A. thaliana* transgenic lines, OE1 and OE2, which overexpress PgCAMTA3, were generated through genetic transformation. The overexpression of *PgCAMTA3* enhanced the cold stress tolerance in transgenic *A. thaliana*. OE1 and OE2 exhibited higher survival rates under cold stress. Furthermore, enzymatic activity assays revealed enhanced peroxidase (POD), catalase (CAT), and superoxide dismutase (SOD) in OE lines. These antioxidant enzymatic activities collectively contribute to better cold stress tolerance by providing more effective reactive oxygen species (ROS) scavenging and cellular protection mechanisms, which was confirmed by lower levels of malondialdehyde (MDA) and ROS production. In addition, the overexpression of *PgCAMTA3* led to the upregulation of the expression levels of *AtCBF2*, *AtNCED3*, and *AtWRKY22*, which were modulated by *CAMTA3*. In summary, we report the significant role of *PgCAMTA3* in plant cold tolerance. Our findings provide valuable insights into the CAMATA family in plants and offer new perspectives on the molecular mechanisms underlying cold tolerance in pomegranates.

## 1. Introduction

Abiotic stresses, including cold, drought, and high salinity, represent significant environmental challenges for plants. These stresses adversely affect plant growth and development, leading to reductions in crop yields and quality [1,2]. Among these, cold stress, comprising both freezing stress (<0 °C) and chilling stress (0~15 °C), is a key ecological factor that limits plant growth and determines plants’ geographical distribution [3]. Exposure to cold stress induces a range of morphological, physiological, and metabolic changes in plants [4,5]. In response to cold stress, many plants, including *Arabidopsis thaliana*, *Brassica napus*, and *Triticum aestivum*, have evolved complex adaptive mechanisms that involve a wide range of physiological, biochemical, and metabolic changes [5,6]. These adaptations include the accumulation of metabolites, alterations in lipid composition, and the regulation of cell membrane integrity, the cell wall structure, and cytoskeletal stability [3,4,5,6].

Molecular regulation plays a critical role in cold stress response, involving stress perception, signal transduction, gene expression, and cellular repair [2,4]. Numerous genes are involved in the complex molecular networks that regulate cold stress response. Among these, certain genes are engaged in signal reception and transduction, regulating the expression of downstream genes that modulate the overall cold stress response [4,5,6].

Calcium, as an intracellular second messenger, plays crucial roles in various signal transduction pathways [7]. Under stress stimuli, the intracellular [Ca^2+^]_cyt_ level increases rapidly. The calcium-binding protein calmodulin (CaM) senses the changes in [Ca^2+^]_cyt_ and transmits the signal to the downstream components [8]. CAMTA, a signal-responsive protein that is widely present in eukaryotes, functions as a transcription factor that is involved in regulating plant stress responses [9]. CAMTA structures are conserved and include an ankyrin (ANK) repeat domain, an IQ (isoleucine glutamine) domain, a CaM-binding domain, and a CG-1 DNA-binding domain [10]. The IQ domain and the CaM-binding domain bind to CaM in a calcium-dependent manner, suggesting that the functional activation of CAMTA depends on the binding of calcium ions/CaM [11]. In addition to these more conserved domains, some CAMTA proteins contain a TIG (transcription-associated immunoglobulin-like) domain, while others do not [12]. The *A. thaliana* CAMTA family includes six members, AtCAMTA1–6. AtCAMTA1, AtCAMTA2, and AtCAMTA3 do not contain the TIG domain, whereas AtCAMTA4, AtCAMTA5, and AtCAMTA6 do. The TIG domain typically functions in non-specific DNA binding. The CG-1 DNA-binding domain is located at the N-terminus of the CAMTA protein, which enables the CAMTA protein to specifically bind to target genes [13]. CAMTA proteins regulate the transcriptional expression of target genes by specifically binding to the *cis*-acting elements (A/C/G)CGCG(T/C/G) or (A/C)CGTGT in the promoter regions of target genes [10,11]. ACGTGT is also responsive to abscisic acid (ABA) [14,15].

In *A. thaliana*, there are six CAMTA proteins that respond rapidly and differentially to various environmental stresses [16]. Among the AtCAMTAs, AtCAMTA1 and AtCAMTA3 have been studied for their responses under cold stress, and AtCAMTA3 has been extensively studied [17]. AtCAMTA1 is also associated with drought stress responses through the ABA-dependent pathway [18]. In *camta3* mutant lines, the expression of *CBF2* decreased by 50% compared to wild-type plants under cold stress [17]. CBF (C-repeat binding factors) proteins, also referred to as DREB1 (dehydration-response element-binding factors), have been identified as crucial transcription factors that play a pivotal role in the response to cold stress [19,20]. CBF proteins belong to the family of AP2/ERF (APETALA2/ethylene response element-binding factor) transcription factors [21]. CBF family members, *CBF1–3*, are rapidly induced by cold stress. The *cbf1/2/3* triple mutant displays hypersensitivity to freezing stress under cold-acclimation treatments. The overexpression of *CBFs* enhances freezing tolerance in many plant species [20,22]. Thus, CAMTA3 acts as a positive regulator for plant responses to cold. Apart from *A. thaliana*, CAMTAs from other plant species have also been studied in terms of their abiotic stress responses. For example, researchers have analyzed the expressions of CAMTAs in *Gossypium hirsutum* and *T. aestivum* CAMTAs under heat stress, *Glycine max* CAMTAs in drought stress, *Linum usitatissimum* CAMTAs in light responses and low temperatures, and *Phoebe bournei* CAMTAs in heat and drought [23,24,25,26,27].

Pomegranate (*Punica granatum* L.) is often referred to as a ‘miracle fruit’ due to its high nutritional value, ecological importance, and medicinal properties. It is widely cultivated in countries such as China, India, Spain, the United States, and Iran [28,29,30]. However, cold stress has emerged as a significant limiting factor for the pomegranate industry, as pomegranate plants cannot tolerate prolonged exposure to temperatures below −15 °C [31,32]. Most research on pomegranate cold tolerance has focused on preserving the fruit during refrigeration, while less is known about the molecular mechanisms underlying cold resistance during the plant’s growth stages. With the rapid development of plant genome sequencing, the whole-genome identification of the CAMTA family has been conducted in various species including *A. thaliana*, *T. aestivum*, *G. max*, *Zea mays*, and *Oryza sativa* [24,33,34,35]. Nevertheless, studies of the CAMTA family in pomegranate are lacking, and the composition of CAMTA and its roles in pomegranate remain poorly understood. In order to identify the mechanism of cold stress responses in pomegranates, it is imperative to identify and characterize functions of key genes related to cold tolerance. In the present study, we performed a comprehensive analysis of the CAMTA family in pomegranates using bioinformatic methods. Pomegranate *CAMTA3* (*PgCAMTA3*) was identified and characterized to enhance cold resistance in transgenic *A. thaliana* lines that overexpress *PgCAMTA3*. In pomegranates, the expression of *PgCAMTA3* is induced by ABA and cold treatment. Furthermore, enzymatic activity assays for peroxidase (POD), catalase (CAT), and superoxide dismutase (SOD) confirmed the superior performance of the *PgCAMTA3*-OE lines. In addition, the overexpression of *PgCAMTA3* led to the upregulation of the expression levels of *AtCBF2*, *AtNCED3*, and *AtWRKY22* in the transgenic *A. thaliana* lines. We also identified *PgCBF2*, Pg*NCED3*, and Pg*WRKY22* and found that their promoter regions contain *cis*-elements for CAMTA3 binding, indicating that they are candidate genes modulated by PgCAMTA3.

## 2. Results

### 2.1. Identification of CAMTA Genes in Pomegranate

A total of 79 *CAMTA* genes were identified in *P. granatum* (pomegranate) and 11 other plant species (including 15 in pomegranate, 6 in *A. thaliana*, 7 in *G. hirsutum*, 9 in *G. max*, 5 in *T. aestivum*, 10 in *A. hypogaea*, 4 in *O. sativa*, 5 in *Z. mays*, 9 in *S. lycopersicum*, 3 in *S. bicolor*, 3 in *T. plicata*, and 3 in *S. moellendorffii*). The number of amino acids, the molecular weight (MW), and the isoelectric point (pI) were computed based on the predicted protein sequences of the identified *CAMTA* genes. As shown in Table S1, the lengths of the predicted CAMTA proteins ranged from 501 to 1143 amino acids, with relative molecular weights from 55.54 kDa to 133.87 kDa, and theoretical pIs ranging from 4.99 to 9.15. The subcellular localization of these genes was predicted, showing that the majority of the CAMTA proteins in plants are located in the nucleus, with some in the cytoplasm and mitochondria.

### 2.2. Phylogenetic Analysis of CAMTA Genes

**Figure 1:**
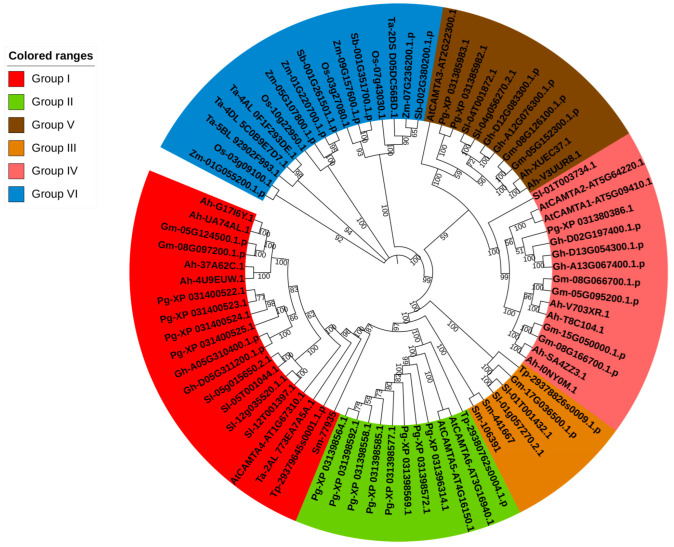
Phylogenetic tree of the CAMTA proteins from 12 plant species. Different colors represent the six different groups of CAMTAs. At: *A. thaliana*, Os: *O. sativa*, Pg: *P. granatum e*, Gh: *G. hirsutum*, Gm: *G. max*, Ta: *T. aestivum*, Ah: *A. hypogaea*, Za: *Z. mays*, Sl: *S. lycopersicum*, Sb: *S. bicolor*, Tp: *T. plicata*, and Sm: *S. moellendorffii*.

To investigate the evolution of homologous *CAMTA* genes, we constructed phylogenetic trees using CAMTA protein sequences from *A. thaliana*, *O. sativa*, pomegranate, *G. hirsutum*, *G. max*, *T. aestivum*, *A. hypogaea*, *Z. mays*, *S. lycopersicum*, *S. bicolor*, *T. plicata*, and *S. moellendorffii* using the maximum likelihood (ML) method. A total of 79 identified CAMTA proteins from these 12 species were categorized into six distinct groups based on the phylogenetic analysis. Notably, species that are more closely related exhibited greater similarity in protein classification. In *A. thaliana*, six CAMTA proteins were classified into groups I, II, IV, and V. Groups I and VI contained a higher number of proteins than the other groups. In pomegranate, CAMTA proteins were present in all groups except groups III and VI (Figure 1).

### 2.3. Gene Structure Analysis of the CAMTA Genes in Pomegranate

**Figure 2:**
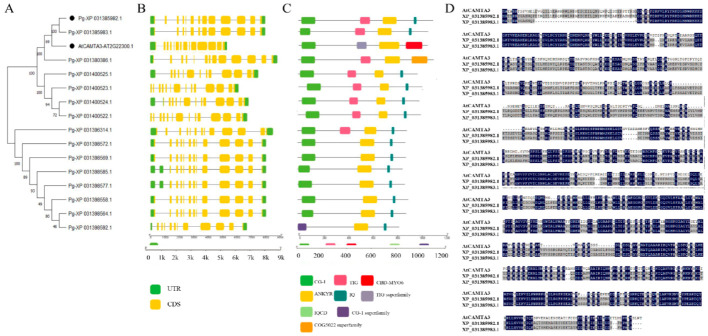
Conserved domains and motifs of AtCAMTA and PgCAMTA proteins. (**A**) Phylogenetic analysis of *AtCAMTA3* and *PgCAMTA* genes. (**B**) The gene structure of *AtCAMTA* and *PgCAMTA* genes. UTR (untranslated region), CDS (coding sequence), and introns are represented by green boxes, yellow boxes and black single lines, respectively. (**C**) Distribution of conserved motifs in AtCAMTA3 and PgCAMTA proteins. The eight motifs are represented by different colored boxes. (**D**) Protein sequence alignments of XP_031385982.1 and XP_031385983.1 with AtCAMTA3. The colored regions represent conserved amino acid sequences.

Gene expression and function are closely related to gene structure. To investigate the structural characteristics of CAMTA proteins, we analyzed the sequence features of the CAMTA family in pomegranate. Specifically, we examined the exon–intron structure of these genes. In *A. thaliana*, *AtCAMTA3* contains 13 introns and 14 exons. In contrast, two *CAMTA* genes in pomegranate contain 11 introns, while others had 12 or 13 introns (Figure 2B).

We used the CD search tool from NCBI to identify common motifs in PgCAMTA proteins, highlighting nine highly enriched motifs, as shown in Figure 2C. The locations and frequencies of these motifs were analyzed in conjunction with the phylogenetic tree results. Three or four motifs were identified in every PgCAMTA protein. All CAMTA proteins contained a CG-1 domain at the N-terminus, which is a pivotal member of a rapid stress response element (RSRE) found in the promoters of many genes that are rapidly activated in response to stress. All CAMTA proteins also contained an ANK ankyrin repeat domain and IQ domain. Only PgCAMTA in group II is missing the TIG domain. This result reflects the conservation of CAMTA in pomegranates.

**Figure 3:**
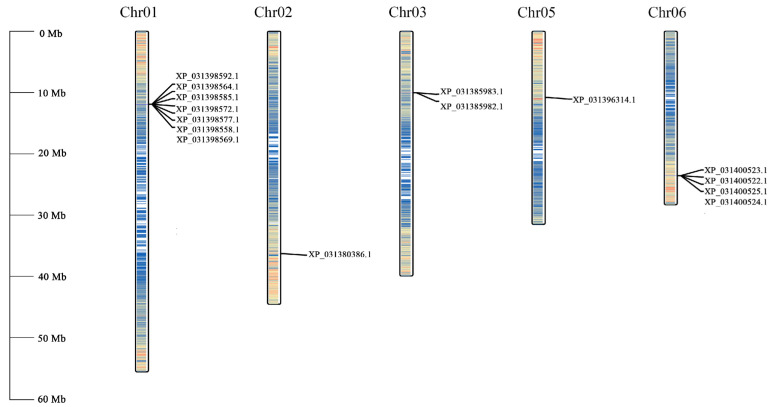
The chromosomal locations of *PgCAMTA* genes from pomegranate.

The 15 *PgCAMTA* genes were distributed across five distinct scaffolds of the genome assembly. The chromosome Chr01 contained the largest number, with seven *PgCAMTA* genes, while Chromosome Chr06 harbored four *PgCAMTA* genes. Two genes were located on chromosome Chr03 NC-045129.1. Chromosome Chr02 and chromosome Chr05 contained single *PgCAMTA* genes (Figure 3).

We analyzed the protein sequences of PgCAMTA, which had the highest homology with AtCAMTA3. XP_031385982.1 and XP_031385983.1 were classified into group V with AtCAMTA3. XP_031385983.1 is shorter than XP_031385982.1 by 40 amino acids (Figure 2D). Therefore, we designated XP_031385982.1 as PgCAMTA3 and used it for the downstream analyses.

### 2.4. Analysis of Cis-Acting Elements in the Promoter Regions of PgCAMTA3 Genes

**Figure 4:**
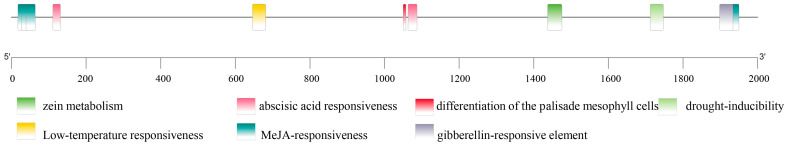
*Cis*-elements in the *PgCAMTA3* promoter region.

Cis-elements play a crucial role in regulating gene expression and influencing plant growth, development, and responses to environmental stresses. To further investigate these *CAMAT* genes, we analyzed the 2000 bp upstream sequence of the *PgCAMTA3* gene using PlantCARE online tool. A variety of *cis*-elements were found in the promoter region of the *PgCAMTA3* gene, including those involved in hormone responses, abiotic stress responses, and transcription factor (TF) binding sites. Specifically, *cis*-elements related to gibberellin, abscisic acid, and methyl jasmonate (MeJA) were identified (Figure 4). Additionally, *cis*-elements associated with low-temperature responses and drought-inducibility defense mechanisms were identified. These findings suggest that the *PgCAMTA3* gene may be involved in multiple stress responses, indicating their potential role in plant adaption to diverse environmental conditions.

### 2.5. The Expression of PgCAMTA3 Genes Under ABA and Cold Stress

**Figure 5:**
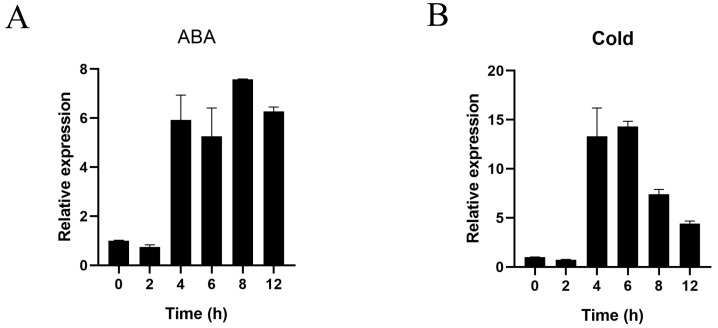
The expression of *PgCAMTA3* is induced by ABA and cold treatment. (**A**) Expression patterns of the *PgCAMTA3* gene under the 100 μM ABA treatment. (**B**) Expression patterns of *PgCAMTA3* under cold treatment. The error bars represent the standard deviations of three technical replicates.

Our phylogenetic analysis showed that *PgCAMTA3* is closely related to other CAMTA genes which are involved in cold stress and ABA responses. To further investigate the role of *PgCAMTA3* under cold stress, we performed a qPCR to measure its expression in pomegranates. Indeed, expressions of *PgCAMTA3* were detected at 0 h, 2 h, 4 h, 6 h, 8 h, and 12 h following cold and ABA treatment. A transient increase in expression was detected 4 h after cold and 100 μM ABA treatment. The expression began to decrease after 8 h of cold treatment but remained high after 12 h of ABA treatment. Notably, *PgCAMTA3* exhibited stronger increase in expression levels in cold stress, suggesting a strong association with cold stress (Figure 5). Based on these findings, we hypothesize that *PgCAMTA3* plays a significant role in the cold stress response.

### 2.6. Overexpression of PgCAMTA3 Enhanced Freezing Tolerance in A. thaliana

**Figure 6:**
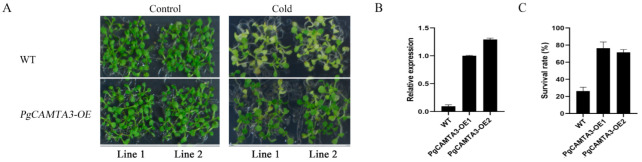
Enhanced cold tolerance in *A. thaliana* transgenic lines overexpressing *PgCAMTA3* genes. (**A**) Wild-type (WT) and *PgCAMTA3* transgenic *A. thaliana* lines with and without cold treatment. (**B**) qPCR analysis of gene expression in WT and *PgCAMTA3* transgenic *A. thaliana* lines. (**C**) Survival rate of wild-type and *PgCAMTA3* transgenic *A. thaliana* lines following cold treatment.

**Figure 7:**
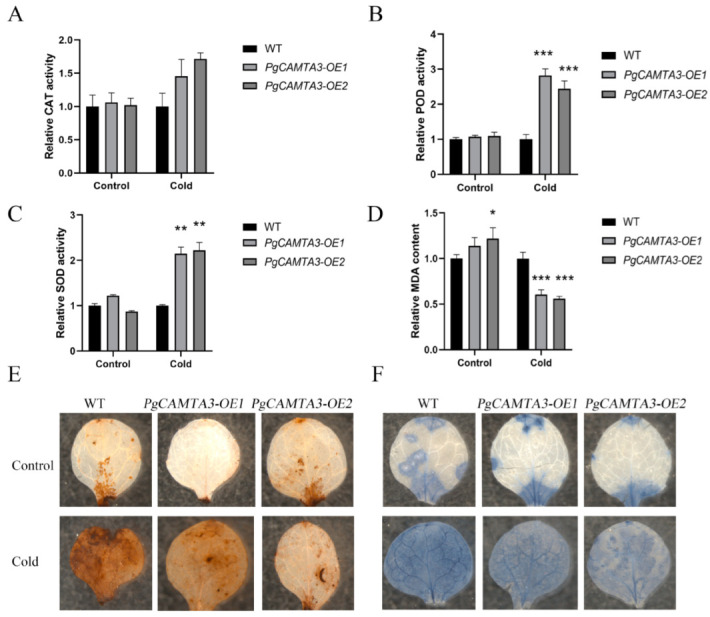
Physiological assays of wild-type (WT) and OE *A. thaliana* lines under cold stress. (**A**–**D**) Enzyme activity of CAT (**A**), POD (**B**), and SOD (**C**), and MDA content (**D**) in wild-type and *PgCAMTA3*-overexpressing transgenic lines. * denotes a significant difference at *p* < 0.05, ** denotes a significant difference at *p* < 0.01, *** denotes a significant difference at *p* < 0.001. (**E**) Detection of H_2_O_2_ accumulation via DAB staining in wild-type and *PgCAMTA3*-overexpressing transgenic lines. (**F**) Detection of O^2−^ accumulation by NBT staining in wild-type and *PgCAMTA3*-overexpressing transgenic lines.

*PgCAMTA3* sequences were cloned from the pomegranate cultivar “Taishanhong”, and transgenic lines *A. thaliana* that overexpress *PgCAMTA3* were generated. We found that the survival rate of the transgenic lines, *PgCAMTA3-OE1* and *PgCAMTA3-OE2*, exceeded 70%, which was higher than the wild-type plants (~25%) following 1 h treatment at −2 °C (Figure 6). To assess the response of the transgenic lines, we investigated various physiological and biochemical parameters in both wild-type and transgenic plants after a six-day treatment at 4 °C. Key indices, including the MDA content and enzyme activity of SOD, POD, and CAT, were measured. The results revealed that SOD, POD, and CAT activities were significantly higher in the *PgCAMTA3-OE* lines, while MDA content was lower (Figure 7A–D). Collectively, these results suggest that the overexpression of *PgCAMTA3* in *A. thaliana* enhanced cold tolerance, possibly through preserving cell membrane integrity and reducing oxidative damage.

Subsequent DAB and NBT staining revealed no significant color difference between transgenic and wild-type plants under normal conditions. However, after cold treatments, the wild-type plants showed more extensive brown or blue staining, indicating higher ROS accumulation (Figure 7E,F). Taken together, *PgCAMTA3* may enhance cold tolerance in the transgenic *A. thaliana* plants via the more efficient scavenging of ROS.

### 2.7. PgCAMTA3 Activated Downstream Genes in Transgenic A. thaliana

**Figure 8:**
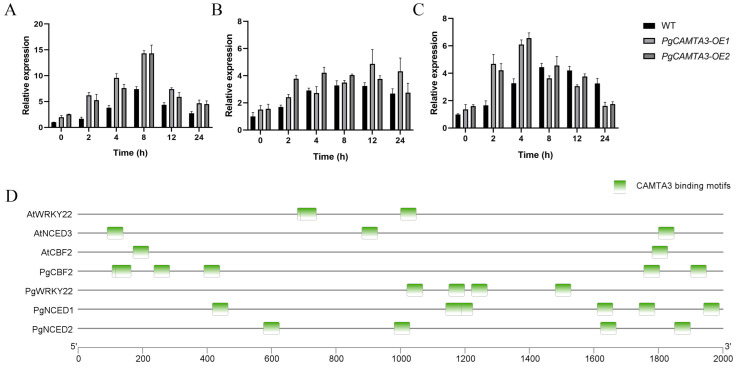
The expression levels of genes related to cold stress in *A. thaliana* under cold treatment. (**A**) The expression levels of *AtCBF2* in WT, *PgCAMTA3-OE1*, and *PgCAMTA3-OE2* under cold treatment. (**B**) The expression levels of *AtWRKY22* in WT, *PgCAMTA3-OE1*, and *PgCAMTA3-OE2* under cold treatment. (**C**) The expression levels of *AtgNCED3* in WT, *PgCAMTA3-OE1*, and *PgCAMTA3-OE2* under cold treatment. (**D**) CAMTA3 binding motifs in the promoter of the target genes *PgCBF2*, *PgNCED1*, *PgNCED2*, and *PgWRKY22*.

In order to determine whether the overexpression of *PgCAMTA3* modulates the genes with which *PgCAMTA3* interacts, we selected *CBF2*, NINE-CIS-EPOXYCAROTENOID DIOXYGENASE 3 (*NCED3*), and *WRKY22* to determine their expressions. The transgenic *A. thaliana* lines were treated with cold stress. We analyzed the expression pattern of the three gene interactors of *PgCAMTA3*. The qPCR result showed that *CBF2*, *NCED3*, and *WRKY22* displayed similar expression profiles in the control and cold-treated groups. All of these genes were upregulated in response to cold, and their expression levels were higher in the *PgCAMTA3* overexpression lines (Figure 8A–C). *AtCBF2*, which has been identified as crucial transcription factor that plays a pivotal role in the response to cold stress, was upregulated, while, in the OE plants, its upregulation was two-fold higher than that in the WT. *NCED3* is known as an important enzyme for ABA accumulation during drought stress and was rapidly induced by cold exposure. *WRKY* regulates plant tolerance to various stresses such as salt, low temperature, drought, and flowering. *AtNCED3* and *AtWRKY22* were also significantly upregulated, which is consistent with *AtCBF2*. In the promoter of *AtCBF2*, *AtNCED3*, and *AtWRKY22*, many *CAMTA* binding sites ((A/C/G)CGCG(T/C/G) or (A/C)CGTGT) were found. Their homologous gene in pomegranates was also found in many *CAMTA* binding sites (Figure 8D).

## 3. Discussion

Cold stress is a severe abiotic stress that significantly affects crop yield [5,36]. For perennial crops such as pomegranate, cold tolerance is crucial for survival during autumn and winter and also determines the quality and yield of the pomegranate fruit [32]. As signal-responsive proteins, *CAMTA* genes have been shown to play a significant role in both biotic and abiotic stresses [9,10,17]. CAMTA proteins bind to the CGCG motif in the promoters of its target genes, while CAMTA3 binds to vCGCGb or vCGC/TGb motifs [37]. *CAMTA3* is involved in drought, cold, and wounding stress responses and plays a significant role in plant growth and development [37]. *CBFs* have a positive regulatory role in plant cold resistance [38,39]. Transgenic plants that overexpress *CBF* genes in species such as rice, soybean, pear, and sweet potato have shown enhanced cold resistance. CAMTA can bind to *CBF* promoters to regulate their expression. Although the CAMTA family has been extensively studied in many plant species, an analysis of the CAMTA family in pomegranate is still lacking. To investigate the relationship between cold tolerance and *CAMTA* genes in pomegranate, we used CAMTA3 from *A. thaliana* for the retrieval and identified members of the CAMTA family in pomegranate and 11 other plant species. Through our queries, we obtained 79 *CAMTA* genes from these species. These genes have different molecular weights and are primarily located in the nucleus. In a previous study, an analysis of the CAMTA family revealed the presence of *CAMTA* genes in *S. moellendorffii*; similarly, our research found *CAMTA* genes in *S. moellendorffii* and *T. plicat*. Notably, the subcellular localization results showed that only two genes were located in the cytoplasm or mitochondria, while the others were in the nucleus, which is consistent with CAMTA’s role as a transcription factor for transcriptional activation.

In previous studies, CAMTA was also found to be located in the nucleus, such as in TgCAMTA1 and TgCAMTA3 in *Tectona grandis* [40] and PpCAMTA5 in peach [41]. But not all transcription factors are located in the nucleus: for instance, bHLH039 [42]. When the iron-induced transcription factor FIT is lacking, bHLH039 is mostly located in the cytoplasm, but, if FIT is present, bHLH039 shows strong nuclear localization. The physical exclusion of transcriptional regulators from the nucleus by sequestration in the cytoplasm is one strategy for regulating their activity, such as the transcription factors BES1, BZR1, and PHR1 [43,44]. Based on software predictions, most PgCAMTA is located in the nucleus. A few CAMTAs exist in the cytoplasm or mitochondria. The matter of how cytoplasmic CAMTA genes perform the function of transcription factors needs further study.

Our phylogenetic analysis revealed that the CAMTA family is divided into six distinct clades. Notably, Clade VI exclusively contains *CAMTA* genes from monocot species such as wheat, maize, rice and sorghum, suggesting that these genes may play specialized roles in monocots. The *CAMTA* genes from pteridophytes and gymnosperms were clustered in Clade III, which may represent the most ancient CAMTA family genes. In addition, within each clade, CAMTAs from the same species showed the closest genetic relationship. Comparing the CAMTA families of pomegranate and *A. thaliana*, we observed that most of these genes have more than 11 introns, but all contain the conserved TIG, CG1 DNA-binding domain, and ANK ankyrin repeat domain. These results showed that CAMTAs contained a very conserved domain.

CAMTAs can sense stress signals from Calmodulin and interact with the stress-responsive genes. Under cold stress, a truncated version of CAMTA3 rapidly induced the expression of *EXPL1*, *CBF2*, and *NCED3*, and it could restore freezing tolerance in a *camta23* double mutants. The WRKY transcription factor family is also closely related to cold stress and can induce the genes regulated by cold stress in a similar manner to CBFs [45,46]. In the *PgCAMTA3-OE* transgenic lines, the expression of cold-stress-related genes was analyzed. The expression of these genes was induced by cold stress, and the expression was higher in the *PgCAMTA3-OE* transgenic lines. The expression of the CBF2 response was the strongest. These results indicate that the enhanced cold tolerance of transgenic plants not only depends on the CBF-dependent pathway but is also involved in the CBF-independent pathway.

To further investigate the functions of *CAMTA* genes, we cloned *PgCAMTA3* in pomegranates with high homology to *AtCAMTA3* for a functional analysis of the response to cold stress. This gene was heterologously expressed in *A. thaliana*, and the transgenic lines demonstrated enhanced cold tolerance. In our study, the *PgCAMTA3-OE* lines exhibited the lowest mortality after cold stress, along with higher SOD, POD, and CAT activities and lower MDA content, indicating better stress resilience. DAB and NBT staining further revealed light staining in the *PgCAMTA3-OE* transgenic lines, indicating lower levels of peroxide accumulation and enhanced abilities to scavenge reactive oxygen species. These finding are consistent with previous studies, confirming the role of *PgCAMTA3* in enhancing cold tolerance.

## 4. Materials and Methods

### 4.1. Plant Material, Growth Conditions

The pomegranate cultivar “Taishanhong” was planted in the Shandong Institute of Pomology of Taian, China. Cuttings from “Taishanhong” were cultured in a growth chamber at 22 °C and 65% humidity under a 14 h/10 h light/dark photoperiod. Tender leaves growing on cuttings were collected for RNA extraction to clone the genes. Additionally, *A. thaliana* seedlings (ecotype Columbia, Col-0) were grown under the same conditions.

### 4.2. Identification of CAMTA Genes

The protein sequences of AtCAMTA3 (AT2G22300.1) were obtained from TAIR webserver (https://www.arabidopsis.org/, accessed on 5 May 2024). The pomegranate genome and CAMTA family and annotation files were downloaded from NCBI (https://www.ncbi.nlm.nih.gov/datasets/genome/GCF_007655135.1/, accessed on 5 April 2024). The CAMTA members of other plant species were obtained via a BLASTP search using AtCAMTA3 as queries on phytozome 13 (https://phytozome-next.jgi.doe.gov/, accessed on 12 May 2024) (E-value ≤ 1 × 10^−90^).

The length of the amino acids, the molecular weight (MW), and the isoelectric point (pI) were calculated using the ExPasy ProtParam tool (http://web.expasy.org/protparam/, accessed on 15 May 2024). BUSCA (http://busca.biocomp.unibo.it/, accessed on 15 May 2024) was employed for the prediction of subcellular localization. PlantCARE (http://bioinformatics.psb.ugent.be/webtools/plantcare/html/, accessed on 20 August 2024) was used to predict the *cis*-elements of CAMTA promoters.

### 4.3. Phylogenetic Analysis

The protein sequences of the identified CAMTA genes were aligned, and the phylogenetic relationships were estimated using the maximum likelihood (ML) method in MEGA 11 with the default parameters. The phylogenetic tree was beautified using the online iTOL program (https://itol.embl.de/, accessed on 15 June 2024).

### 4.4. Prediction of the Motifs/Domains of CAMTA Genes

The MEME program (https://meme-suite.org/meme/tools/meme, accessed on 15 January 2024) was used to analyze the composition of conserved motifs in CAMTA members. Conserved domains were acquired using the CD-search tool on NCBI (https://www.ncbi.nlm.nih.gov/Structure/bwrpsb/bwrpsb.cgi, accessed on 20 January 2024). The gene structures and chromosomal locations were acquired from their genome annotation files. The results were visualized using TBtools-II.

### 4.5. Recombination Vector Construction and Transgenic Plant Generation

We cloned *PgCAMTA3* using the specific primers (Supplementary Table S2) and integrated the generated fragments individually into the p1306-Flag vector, generating the recombinant vector p1306-35S-*PgCAMTA*3 (Figure S1). The *PgCAMTA3* fragment was linked to the p1306-Flag vector via double enzyme digestion. The enzyme sites were *Bam*H I and *Sal* I. The recombinant vectors were introduced into *A. thaliana* (ecotype Columbia, Col-0) via the *Agrobacterium tumefaciens* strain GV3101 using the floral dip method [47]. Hygromycin was used to select positive transgenic seedlings, and their *PgCAMTA*3 expression levels were detected.

### 4.6. Cold Tolerance Assays

Cuttings from “Taishanhong” were subjected to cold treatment at 4 °C and the leaves were cultured in solutions containing 100 μM ABA. Three replicates were collected and immediately frozen in liquid nitrogen at 0 h, 2 h, 4 h, 6 h, 8 h, and 12 h following treatment. The frozen samples were subsequently stored in a −80 °C freezer for total RNA extracted.

Freezing tolerance assays were conducted as described by Hu et al., with some modifications [48]. The 8-day-old *A. thaliana* seedlings were maintained for 1 h in a growth chamber at −2 °C, and then transferred to 22 °C. The survival rates were measured visually after 24 h. The survival rates of each line were calculated based on three replicates. About 30 seedlings were used for each experiment. The 12-day *A. thaliana* plants were divided into two groups, one group (the cold-acclimated group) was set at 4 °C, while the other group was the control. The samples were collected for the qPCR experiment after 0 h, 2 h, 4 h, 8 h, 12 h, and 24 h for RNA extraction.

### 4.7. RNA Isolation and qPCR Analysis

The total RNA of the cuttings and leaves was extracted from the pomegranate leaves using the Plant Total RNA Isolation Kit (Shanghai Sangon Biotech Co., Ltd., Shanghai, China). The qRT-PCR was carried out using ChamQ Universal SYBR qPCR Master Mix (Beijing Tiangen Biotech Co., Ltd., Beijing, China) on an ABI 7500 PCR instrument (Applied Biosystems, Foster, CA, USA). The *PgActin* (LOC116200207) and *AtActin* (AT3G18780) genes were used as an internal reference. The qPCR experiment was repeated three times. All the primers used are listed in Supplementary Table S2. GraphPad Prism 8 software was used for the data analysis and significance tests.

### 4.8. Determination of MDA Content and Enzyme Activities

MDA content was measured using thiobarbituric acid (TBA) reactive through the MDA-2-Y Kit (Suzhou Comin Biotechnology Co., Ltd., Suzhou, China). The superoxide dismutase (SOD) activity was assayed using the nitro blue tetrazolium (NBT) method. The enzyme activities of catalase (CAT) activity were assayed using the ammonium molybdate method. The CAT, peroxidase (POD), and SOD were measured using a CAT-2-W Kit, a POD-2-Y Kit, and a SOD-2-Y Kit (Suzhou Comin Biotechnology Co., Ltd., Suzhou, China), respectively [49,50]. GraphPad Prism 8 software was used for the data analysis and significance tests.

### 4.9. Histochemical Staining

We used 3,3-diaminobenzidine (DAB) and nitroblue tetrazolium (NBT) staining to detect hydrogen peroxide (H_2_O_2_) and superoxide anions (O_2_^−^), respectively, as described in the previous study [51].

## 5. Conclusions

We investigated the *CAMTA* family in 12 plant species, including pomegranate, using bioinformatic methods. A total of 79 CAMTA proteins were found. Transgenic *A. thaliana* plants overexpressing *PgCAMTA3* were used to investigate their potential functions under cold stress. The *PgCAMTA3-OE* transgenic lines had a higher survival rate, along with higher POD, CAT, and SOD activities and lower MDA content after the cold treatment, suggesting that *PgCAMTA3* enhanced cold tolerance in transgenic *A. thaliana* plants under cold stress. In the transgenic lines, *CBF2*, *NCED3*, and *WRKY22* were activated. It is possible that CAMTA3 and CBF2, NCED3, WRKY22 synergistically participate in cold stress responses. Our findings provide new insights for researching the cold tolerance mechanisms of pomegranate.

## Data Availability

Data is contained within the article or Supplementary Materials.

## Supplementary Materials

The following supporting information can be downloaded at: https://www.mdpi.com/article/10.3390/plants14050813/s1. Table S1: Summary of *CBF* genes detected in plant species. Table S2: Sequences of primer pairs used in this study. Figure S1: The vector map of p1306-35S-*PgCAMTA*3.
