# Supplementary material for: Functional Characterization of Pomegranate CAMTA3 in Cold Stress Responses
Source: Plants (Basel). 2025 Mar 5;14(5):813. doi: 10.3390/plants14050813 (PMC11901912; doi:10.3390/plants14050813)
Supplement: Supplementary file 1 [file plants-14-00813-s001.zip › plants-3483874-supplementary.pdf]

Supplementary Materials

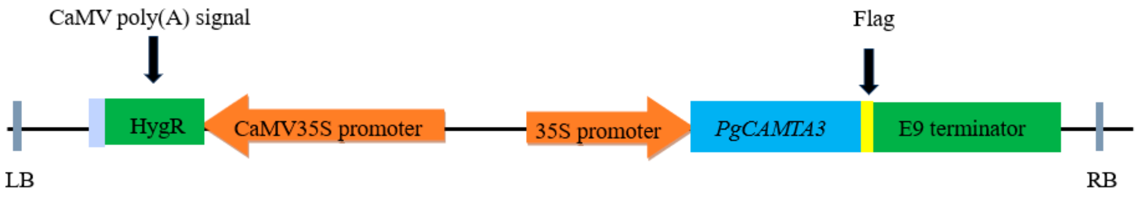

Figure S1 the vector map of p1306-35S-*PgCAMTA*3.

Table S1. Summary of *CAMTA* genes detected in plant species.

Species	Gene ID	No. of	MW	pI	Subcellular Location
S.moellen dorffii	441667	982	109.75	6.18	Nuclear
	106391	625	69.75	9.15	Nuclear
	77935	543	61.56	9.03	Cytoplasmic,Mitochondrial
T.plicata	29378826s0009.1.p	1200	133.87	5.95	Nuclear
	29379645s0001.1.p	1143	128.52	5.59	Nuclear
	29380762s0004.1.p	1070	122.03	6.54	Nuclear
Z.mays	07G236200.1.p	1028	114.07	5.66	Nuclear
	01G220700.1.p	1017	113.04	5.68	Nuclear
	05G107800.1.p	1020	113.25	5.51	Nuclear
	09G157600.1.p	1033	115.46	5.95	Nuclear
	01G055200.1.p	1025	114.42	5.99	Nuclear
T.aestivu m	5BL_92902F993.1	835	93.54	6.07	Cytoplasmic,Nuclear
	4AL_0F1F293DE.1	501	55.62	6.66	Cytoplasmic,Nuclear,Mitochondr
	4DL_5C0B9E7D7.1	501	55.54	6.34	Cytoplasmic,Nuclear,Mitochondr
	2DS_D05DC56BD.1	590	66.01	4.99	Nuclear
	2AL_773EA7A5A.1	1094	121.31	5.57	Nuclear
S.bicolor	001G261501.1.p	1022	113.53	5.63	Cytoplasmic,Mitochondrial
	001G351700.1.p	1024	114.4	5.86	Nuclear
	002G380200.1.p	1030	114.87	5.55	Nuclear
O.sativa	Os03g09100.1	1029	115.01	5.63	Nuclear
	Os10g22950.1	1023	114.12	5.76	Nuclear
	Os07g43030.1	1026	114.67	6.24	Nuclear
	Os03g27080.1	545	61.26	7.25	Nuclear
	04g056270.2.1	1021	114.15	5.76	Nuclear
	01g057270.2.1	957	107.79	8.92	Cytoplasmic,Nuclear,Mitochondr

S.lycopersicum	12g035520.1.1	973	108.98	6.25	Nuclear
	05g015650.2.1	935	105.31	5.89	Nuclear
	04T001872.1	1122	125.07	5.53	Nuclear
	01T003734.1	1066	120.4	5.47	Nuclear
	01T001432.1	945	106.41	8.95	Cytoplasmic,Nuclear,Mitochondr
	05T001044.1	950	106.78	5.92	Nuclear
	12T001397.1	970	108.64	6.19	Nuclear
pomegranate	XP_031380386.1	1083	120.98	5.64	Nuclear
	XP_031385982.1	1078	120.82	5.55	Nuclear
	XP_031400525.1	957	106.64	5.55	Nuclear
	XP_031396314.1	892	100.95	7.02	Nuclear
	XP_031398564.1	886	100.48	6.38	Nuclear
	XP_031385983.1	1038	115.95	5.45	Nuclear
	XP_031400524.1	967	107.67	5.48	Nuclear
	XP_031400523.1	995	111.07	5.47	Nuclear
	XP_031400522.1	1007	112.41	5.47	Nuclear
	XP_031398592.1	830	94.08	6.54	Nuclear
	XP_031398585.1	858	97.36	6.56	Nuclear
	XP_031398577.1	875	99.32	6.85	Nuclear
	XP_031398572.1	880	99.99	6.67	Nuclear
	XP_031398558.1	903	102.44	6.59	Nuclear
	XP_031398569.1	884	100.46	6.67	Nuclear
G.max	05G095200.1.p	1088	121.94	5.58	Nuclear
	05G152300.1.p	1122	126.99	5.79	Nuclear
	05G124500.1.p	983	109.31	5.43	Nuclear
	08G126100.1.p	1102	124.43	5.66	Nuclear
	15G050000.1.p	1088	122.46	5.69	Nuclear
	08G066700.1.p	1079	121.26	5.72	Nuclear
	08G166700.1.p	1085	122.08	5.65	Nuclear
	08G097200.1.p	966	107.89	5.81	Nuclear
	17G036500.1.p	999	112.28	6.81	Nuclear
G.hirsutum	A13G067400.1.p	1067	120.02	5.8	Nuclear
	D13G054300.1.p	1067	119.8	7.74	Nuclear
	A12G076300.1.p	1070	121.66	6.17	Nuclear
	D12G083300.1.p	1088	123.13	6.04	Nuclear
	D02G197400.1.p	1051	118.33	5.67	Nuclear
	D05G311200.1.p	996	111.04	5.63	Nuclear
	A05G310400.1.p	986	109.85	5.61	Nuclear
Arabidopsis	AT5G09410.1	989	111.99	5.89	Nuclear
	AT5G64220.1	1050	117.26	5.99	Nuclear
	AT2G22300.1	1032	116.11	5.36	Nuclear

	AT1G67310.1	1016	113.06	5.33	Nuclear
	AT4G16150.1	923	104.89	6.94	Nuclear
	AT3G16940.1	845	96.18	8.09	Cytoplasmic,Nuclear
A.hypogaea	4U9EUW.1	974	109.09	5.74	Cytoplasmic,Nuclear
	XUEC37.1	1061	121.14	5.55	Nuclear
	V3UUR8.1	1061	121.4	5.55	Nuclear
	I0NY0M.1	1076	120.87	5.67	Nuclear
	V703XR.1	1080	121.22	5.45	Nuclear
	T8C104.1	1080	121.2	5.54	Nuclear
	UA74AL.1	908	99.47	5.54	Nuclear
	37A62C.1	1010	113.37	5.66	PlasmaMembrane,Cytoplasmic,
	SA4ZZ3.1	1076	120.84	5.65	Nuclear
	G17I6Y.1	909	99.49	5.62	Nuclear

Table S2. Sequences of Primmer pairs were used in this study

Genes	Primer-F	Primer-R	
PgCAMTA3	CATTGCTACTATGCTCACGGA	CAGGTCCAGTTTCTTGGGAAT	Primmer Pairs for qPCR
AtCBF2	TCTGAAATGTTTGGCTCCG	ACTCACACACCCACTTACCG	
AtNCED3	GTTTCTGGGAGATGGCTTG	AATGGCGGGAGAGTTTGA	
AtWRKY22	CGGACGACAAAGTAATGCC	AGCAGAGAGAGAAAGAGGACG	
PgACTIN	AGTCCTCTTCCAGCCATCTC	CACTGAGCACAATGTTTCCA	
AtACTIN2	GGCAAGTCATCACGATTGG	CAGCTTCCATTCCCACAAAC	
PgFL-CAM3	CGCGGATCCATGGCGGACACTCGGAGATA	ACGCGTCGACCGATGTCATGGGCATAAATGA	Primmer pair for cloning *PgCAMTA3*
